# Composition of Challenge Substance in Standardized Antimicrobial Efficacy Testing of Wound Antimicrobials Is Essential to Correctly Simulate Efficacy in the Human Wound Micro-Environment

**DOI:** 10.3390/biomedicines10112751

**Published:** 2022-10-29

**Authors:** Anna-Lena Severing, Mia Borkovic, Ewa K. Stuermer, Julian-Dario Rembe

**Affiliations:** 1 Department of Dermatology, University Hospital Düsseldorf, Heinrich-Heine-University Düsseldorf (HHU), Moorenstrasse 5, 40225 Düsseldorf, Germany; 2 Department of Vascular Medicine, University Heart & Vascular Center (UHZ), University Medical Center Hamburg-Eppendorf (UKE), Martinistraße 52, 20251 Hamburg, Germany; 3 Department of Vascular and Endovascular Surgery, University Hospital Düsseldorf, Heinrich-Heine-University Düsseldorf (HHU), Moorenstrasse 5, 40225 Düsseldorf, Germany

**Keywords:** antimicrobials, antiseptics, wound infection, organic challenge, wound exudate, DIN-EN-13727

## Abstract

Current standards insufficiently acknowledge the influence of the wound micro-environment on the efficacy of antimicrobial agents. To address this, octenidine/phenoxyethanol, polyhexanide, povidone-iodine, and sodium-hypochloride/hypochlorous acid solutions were submitted to standard-based (DIN-EN-13727) or modified peptide-based challenges and compared to a simulated clinical reference using human acute or chronic wound exudate (AWF/CWF). Antimicrobial efficacy against *S. aureus* and *P. aeruginosa* was compared using a quantitative suspension method. Agreement between methods were investigated using Bland-Altman (B&A) analysis. Different substances and challenges demonstrated diverging results, depending on class and concentration of agent and challenge. Highly concentrated antiseptics maintained a high efficacy under complex challenges, while especially chlorine-based irrigation solutions showed a remarkably reduced antimicrobial effect. Composition of challenge substance proved more relevant than pure concentration. Therefore, the current standard challenge conditions did not adequately reflect the wound micro-environment with over- or under-estimating antimicrobial efficacy, whilst the modified peptide-challenge showed a higher level of agreement with simulated realistic conditions (AWF/CWF). The results emphasize that a “one-fits-all” approach is not feasible to generalize antimicrobial efficacy, as certain aspects of the complex micro-environment pose a differing influence on varying agents. Based on these results, revision and target focused adaptation of the current standards should be considered.

## 1. Introduction

Antimicrobial irrigation solutions and local antiseptics represent a fundamental part in the armamentarium of handling infection, colonization, and biofilm burden in acute and chronic wounds. Development of modern antimicrobial agents in wound management has contributed to decreasing general occurrence and severity in infectious wound complications. With about 78% of chronic wounds being burdened with biofilm [1] and postoperative wound infection rates still ranging up to 30% depending on type of surgery and location [2], best informed and ideally evidence-based local treatment is crucial.

For clinicians to administer the best local antimicrobial treatment, efficacy dynamics, risk-benefit-profiles, and potential confounding factors for antiseptic agents need to be investigated. These baseline data foster informed decisions and the application of antimicrobial agents in correct indications. Therefore, national and international standards have been introduced to test antimicrobial and antiseptic agents for specific indications in terms of effect and cytotoxicity on an in vitro level to establish baseline efficacy profiles. For wound antiseptics, especially in European countries, the DIN-EN-13727 has become the threshold and most used standard [3]. However, there are several shortcomings to the standard, that cause recent discussions regarding its feasibility for comprehensive and sufficient baseline testing in agent, specifically for the indication as a wound antimicrobial [3,4]. First, the standard was not designed for the context of wounds, but for intact skin antisepsis and efficacy of antiseptic surgical hand disinfection. Additionally, different test settings are possible within DIN-EN-13727, which leads to diverging results of one tested substance and classifies it as more or less antimicrobial effective. Furthermore, relevant wound specific challenges, such as biofilm formation, local compromise of regenerative cells in a chronic wound, and interference of the local wound micro-environment with the substance’s efficacy are not accounted for. While biofilm formation and cell regeneration may be better addressed in subsequent, more advanced test scenarios of the evaluation process, the basic interaction of a substance with the local environment in terms of potential efficacy loss is crucial. Dubbed the “protein error” or “dirty conditions”, the standard attempts to address the matter using bovine albumin and/or sheep erythrocytes in varying combinations and concentrations to simulate conditions in real life. As correct in itself to use challenges for estimating the agent’s performance in a real-world scenario, recent publications have demonstrated significant and relevant variations between standard conditions, challenged conditions, artificially constructed wound conditions, and conditions using human material to simulate the acute/chronic wound micro-environment [3,4,5,6,7]. This leads to incoherent reports and unclear efficacy profiles regarding antimicrobial agents intended for wound irrigation or antisepsis and false translation into subsequent steps of evidence-building for clinical recommendations. Moreover, it raises the relevant demand for close-to-reality test scenarios and challenge substances as well as the question of which test conditions best reflect the clinical setting of a challenging wound micro-environment.

To address this question, different potential simulated test settings, challenge substances, and conditions need to be evaluated. In the past, some studies addressed this by investigating several potential challenge substances (including bovine albumin and sheep erythrocytes) and their feasibility, which led to the standards currently in use [8,9]. However, the use of human material as challenge substance and comparator has only recently been introduced as a translational approach to approximate the human acute and chronic wound micro-environment [3,4,5,10].

To the knowledge of the authors, no study to date performed a broad and comprehensive in vitro comparison between varying challenge conditions and the simulated wound environment. Therefore, the presented work investigated the influence of current standard test conditions, a modified peptide-based (rather than macro-protein) challenge, and the simulated acute/chronic wound conditions (represented by acute or chronic wound exudate) on the efficacy of most used antimicrobial and antiseptic solutions. Evaluated agents included octenidin-dihydrochloride, povidone-iodine, polyhexanide, and different formulations of hypochlorous solutions. The intention was to determine which form of challenge most likely mimics the performance to be expected in a real-world scenario.

## 2. Materials and Methods

### 2.1. Microbial Strains and Cultivation

Antimicrobial efficacy testing was performed using *Staphylococcus aureus* (DSM-799) and *Pseudomonas aeruginosa* (DSM-939, both DSMZ, Braunschweig, Germany) as pathogens. Bacterial strains were cultivated in sterile casein/soy peptone broth (TSB; 15 g/L casein peptone, 5 g/L soy peptone, and 5 g/L sodium chloride) and plated on casein/soy peptone agar plates (TSA). The pH value of TSB and TSA was adjusted to 7.2 using 5 M sodium hydroxide (all AppliChem, Darmstadt, Germany). Both strains were freshly prepared from cryocultures and subcultured twice before usage in assays.

### 2.2. Antimicrobials and Antiseptics

In this study, six antimicrobial and/or antiseptic wound irrigation solutions with different active agent compositions were evaluated regarding their antimicrobial efficacy (Table 1). In addition to the antiseptic reference solutions Octenisept^®^ (0.1% octenidine dihydrochloride and 2% 2-phenoxyethanol; OCT/PE) and Betaisodona^®^ (10% povidone-iodine; PVP-IOD), four antimicrobial irrigation solutions were tested: Lavasorb^®^ (0.40 g/L polyhexanide, 0.02 g/L macrogolum 4000; PHMB) and the three chlorine-based and -releasing agent solutions ActiMaris^®^ forte (0.2% sodium hypochlorite (NaOCl)/3% sea salt; NaOCl/SS, Lavanox^®^ (<0.08% NaOCl; NaOCl), and Granudacyn^®^ (<0.005% NaOCl and HOCl each; NaOCl/HOCl).

### 2.3. Collection and Work-Up of Human Acute and Chronic Wound Exudate (AWF/CWF)

For simulating the human wound micro-environment within the standard-based antimicrobial efficacy test setup, AWF or CWF were added as physiological challenge substances. To minimize the patient-specific effects of the individual samples, a pool of six AWF and 31 CWF patient-samples, respectively was created. Ethical approval for the collection and usage of human acute and chronic wound exudate (AWF and CWF) was obtained by the local ethics committee of Witten/Herdecke University (No. 11/2018). In addition, all patients signed an informed consent. Inclusion criteria for the collection of the used CWF were chronic wounds of any entity with a duration of >12 weeks. In the study, modern wound care was performed according to standard clinical practice guidelines, without changes to the current treatment regimen. Patient demographics are detailed in Table 2.

Before CWF sample collection, wounds were cleaned mechanically with sterile gauze (avoiding bleeding) followed by an irrigation with 5 mL 0.9% NaCl solution or sterile aqua. To avoid falsification of the results, the application of antimicrobials or antiseptics as well as any form of debridement was avoided prior to sampling.

Acute wound exudate (AWF) from six different patients was collected in subcutaneous redon drains after surgical procedure. Since postoperative bleeding may increase within the first hours after surgery, fluid drained during the first 12 h post-surgery was discarded to avoid contamination of the AWF with blood components. Therefore, exudate drained during the following 24 h was collected and transferred immediately to −20 °C for subsequent processing.

Upon experimental usage, collected AWF samples were slowly thawed on ice, each transferred to a 50 mL falcon tube, mixed for 20 s, followed by a centrifugation step (2200× *g*; 15 min) for debris and bacterial pelletization. The supernatant was pipetted to a new 50 mL tube and centrifuged again (2200× *g*; 15 min), while the pellet was discarded. After centrifugation, the resulting supernatant was sterile filtered (0.45 and 0.22 µm filter; both Sarstedt AG & Co. KG, Nümbrecht, Germany). Subsequently, a pool of the six sterile AWFs was prepared, aliquoted into appropriate volumes (600 µL), and stored at −80 °C until usage.

Chronic wound exudate (CWF) was collected from a total of 31 patients using sterile flocked swabs with a tip of nylon fibers (FLOQSwab^®^; COPAN Diagnostics Inc., Murrieta, CA, USA), whereas 3–6 swabs per wound with an average volume of ~145 µL exudate per swab could be obtained. Each CWF soaked swab was transferred to a tube containing 250 µL of 0.9% NaCl and the resulting ~37% CWF-NaCl-solution was immediately stored at −80 °C.

For CWF pool preparation, the first CWF-NaCl-samples were slowly thawed on ice and the swabs were placed in a 50 µm cell strainer (fixed on a 50 mL tube) for centrifugation at 2200× *g* to recover the maximum volume wound fluid of the swab. The obtained exudate was combined with the CWF-NaCl-solution of the respective swab. CWF samples of all 31 patients were pooled, mixed for 20 s, and centrifuged (2200× *g*; 15 min). After sterile-filtration (0.45 and 0.22 µm filters; both Sarstedt AG & Co. KG, Nümbrecht, Germany), the obtained CWF pool was aliquoted (600 µL) and stored at −80 °C until usage.

### 2.4. Challenge Conditions

In addition to the standard challenge conditions (low and high burden) of the DIN-EN-13727, the antimicrobial efficacy was tested under the influence of different molecular (peptide/protein challenges) and physiological (wound challenges) burdens. Table 3 provides an overview of the different combinations of bacterial suspensions (BS) and challenge conditions used in the QSM.

On the one hand, antimicrobial test solutions were evaluated strictly in accordance with DIN-EN-13727, using low burden conditions (LB; 0.3% bovine serum albumin (BSA); SERVA Electrophoresis GmbH, Heidelberg, Germany) and high burden conditions (HB; 3% bovine serum albumin and 3% sheep erythrocytes (SE; Acila Dr. Weidner GmbH, Weiterstadt, Germany) as specified in the standards [8]. Experiments were conducted using a bacterial suspension prepared in diluent solution (DS; 1 g/L casein peptone and 8.5 g/L sodium chloride).

To simulate the micro-environment of acute and chronic wounds with the complex influence of the micro-milieu compared to standardized challenge conditions as outlined from the standards, human acute (AWF) and chronic wound exudate (CWF) were used as a comparative, physiological “real-world simulation” burden (wound challenges). Exudate samples were prepared as described in the previous paragraph and used rather than the generalized standard burden. Due to sampling circumstances, AWF could be used undiluted, while CWF was diluted due to the sampling process and used in a final concentration of approximately 37% of the physiological concentration in the chronic wound bed.

AWF and especially CWF are inhomogeneous challenge substances representing a milieu with a markedly increased inflammatory and proteolytic profile and varying peptide/protein composition. In particular, due to degradation, denaturation, and proteolytic processes, a higher concentration of peptides compared to macro-proteins and an increased amount of exposed protein residues can be found. Therefore, a defined combination of the protein-containing burden conditions described in the standards (LB and HB) and a peptide-rich bacterial test suspension prepared in TSB (rather than DS) was performed to investigate the influence of a modified peptide-challenge on antimicrobial performance.

### 2.5. Quantitative Suspension Method (QSM)

**Figure 1:**
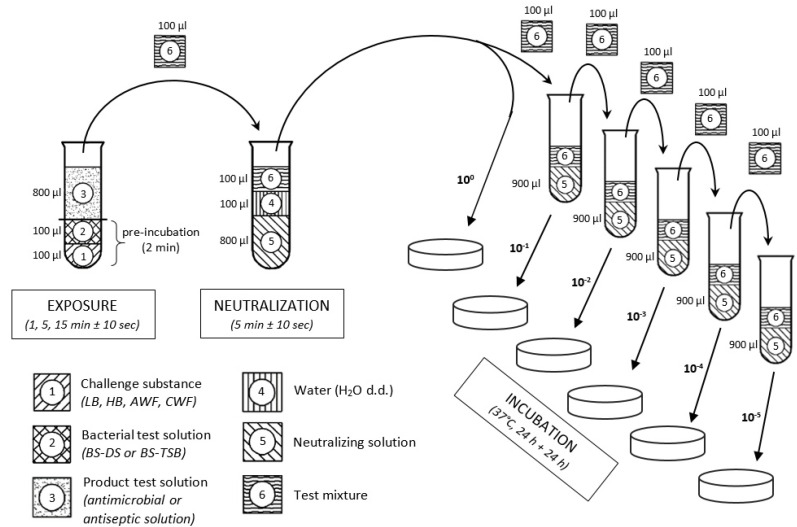
Schematic illustration of the quantitative suspension method based on DIN-EN-13727. Initially, challenge substance (1) and bacterial test solution (2) are pre-incubated together at RT for 2 min. Subsequently, antimicrobial or antiseptic test solution (3) is added for the desired exposure time. Thereafter, a sample of the test mixture (6) is transferred into a neutralizing solution (5) to terminate the antimicrobial effect. Finally, a 10-fold serial dilution series is prepared and samples of each step were plated on agar plates and incubated for at least 24 h [8]. (LB: Low burden; HB: High burden; AWF: Acute wound exudate; CWF: Chronic wound exudate; BS-DS: Bacterial suspension in diluent solution; BS-TSB: Bacterial suspension in TSB).

Antimicrobial efficacy of the antiseptics and antimicrobial solutions was evaluated by quantitative suspension tests based on the dilution-neutralization method of DIN-EN-13727:2009 [8]. Figure 1 illustrates a schematic overview of the used methodology.

Initially, 100 µL of the respective bacterial suspension (BS) based on the dilution solution (BS-DS) or based on the casein/soy peptone broth with higher peptide concentration (BS-TSB) was mixed with 100 µL of the various challenge substances (LB, HB, AWF or CWF) and pre-incubated for 2 min at room temperature (RT). After incubation and mixing, 800 µL of antimicrobial or antiseptic test solution (TS) was added to the bacteria-challenge-mixture and incubated/exposed for 1, 5, and 15 min ± 10 s at RT. To terminate the antimicrobial effect, 100 µL of the test mixture was added to 900 µL of neutralizing solution, which consisted of 3 g/L lecithin, 30 g/L polysorbate 80 (Tween 80), 3 g/L sodium thiosulfate, 10 g/L sodium dodecyl sulfate, and 30 g/L saponin (all Carl Roth GmbH + Co. KG, Karlsruhe, Germany). After 5 min ± 10 s of neutralization, test suspensions were 10-fold serial diluted (10^−1^, 10^−2^, 10^−3^, 10^−4^, and 10^−5^) and 100 µL of each dilution step was plated onto TSA plates, followed by an incubation period of 24–48 h at 37 °C. Subsequently, surviving microorganisms were counted (in cfu/mL) using an automated colony counter (Scan^®^ 500; Interscience, Roubaix, France).

### 2.6. Protein/Peptide Quantification of Challenge Substances

For all challenge conditions, the protein and peptide concentration was measured. The determination in the modified peptide-challenge in bacterial suspension (TSB and DS), the standard burden conditions (LB and HB), and wound challenge (AWF and CWF) were conducted using the Pierce™ BCA protein assay kit (Thermo Fisher Scientific GmbH, Darmstadt, Germany) according to the manufacturer’s instructions. This is a quantitative, colorimetric detection method based on bicinchoninic acid (BCA).

### 2.7. Statistical Analysis

All investigations were performed in duplicates at three to six independent time-points (*n* = 6–12). Data are expressed as mean values ± standard deviation (mean ± SD). Bacterial reduction rates (in Δlog_10_ cfu/mL) were calculated and analyzed using the statistics program GraphPad PRISM (version 9.4.0; GraphPad Software Inc., La Jolla, San Diego, CA, USA). Statistical analysis comprised mixed-effect-models with Dunnett’s post-hoc analysis for multiple comparisons. A value of *p* ≤ 0.05 was considered statistically significant (* *p* ≤ 0.05; ** *p* ≤ 0.01; *** *p* ≤ 0.001; **** *p* ≤ 0.0001).

To investigate the degree of agreement or difference, respectively between the challenge methods used in the norm and modified peptide-based challenges, Bland-Altmann (B&A) analysis was used [11]. AWF and CWF challenges were set as reference standards and the bias between different challenge methods was evaluated as a measure of deviation from the standard. Thereby, the differences between two paired quantitative methods were studied (*y*-axis) and plotted against the average of these measures (*x*-axis) as well as limits of agreement constructed using B&A analysis and plots. The 95% confidence intervals (95% CI) were computed for the bias between two different challenge methods. If two methods compute the exact same quantitative results, no mean bias would be observed from the line of equality (y = 0). A significant systemic difference between the two methods can be observed if the line of equality is not included in the 95% CI of the mean bias. In turn, if the line of equality is included, no significant difference between the methods can be postulated.

## 3. Results

### 3.1. Protein/Peptide Content of Challenge Conditions

**Figure 2:**
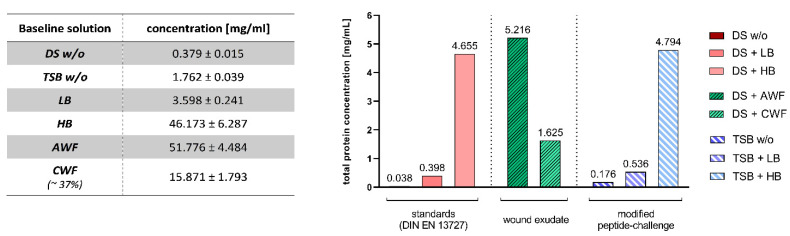
Protein/peptide concentrations in baseline solutions and challenge conditions. Concentrations of the various used baseline solutions (mean ± SD in mg/mL) are summarized in the table (left). Final concentrations (in mg/mL) of the different challenge conditions tested in the experimental qualitative suspension method (QSM) experiments are depicted on the right. Concentrations are sums of the combined baseline solution (dilution solution (DS) or tryptone soy broth (TSB)) and added challenge substance (none—w/o, LB, HB, AWF or CWF). Final concentrations in the experiments are 10-fold diluted due to the experimental setup. Due to the necessary sampling methodology, CWF concentration was diluted to about 37% of physiological concentration in the chronic wound bed. (DS w/o: Dilution solution without additional challenge; TSB w/o: Tryptone soy broth without additional challenge; LB: Low burden; HB: High burden; AWF: Acute wound exudate; CWF: Chronic wound exudate).

Concentrations of the various challenge conditions measured using the colorimetric detection method are summarized and illustrated in Figure 2.

The highest overall protein concentrations in challenge conditions were measured in acute wound exudate (AWF) with 51.16 ± 4.48 mg/mL. Next highest concentrations were measured in the two combinations with an additional high burden (HB; 3% BSA and 3% SE). The standards high burden (DS + HB) showed a concentration of 46.55 ± 6.30 mg/mL and the modified peptide-challenge with a high burden (TSB + HB) 47.94 ± 6.33 mg/mL, respectively.

Chronic wound exudate (CWF) demonstrated a medium overall protein concentration with 15.87 ± 1.81 mg/mL; however, in an already diluted starting condition of 37% (concentration extrapolated to 100%: ~42.89 mg/mL).

The low burden combinations (LB; 0.3% BSA) according to the standards (DS + LB) and the modified peptide-challenge (TSB + LB) showed concentrations of 3.98 ± 0.26 mg/mL and 5.36 ± 0.28 mg/mL, respectively. Finally, the baseline solutions tryptone soy broth (TSB) and dilution solution (DS), in which the bacterial suspension was prepared and tests without challenges were performed, showed the lowest protein concentrations (0.38 ± 0.02 mg/mL for DS and 1.76 ± 0.04 mg/mL for TSB).

### 3.2. Effect of Hypochlorous Wound Irrigation Solutions under Various Challenge Conditions

Chlorine-based and -releasing agents showed differing results regarding antimicrobial efficacy depending on the tested solution, microorganism, and challenge condition.

**Figure 3:**
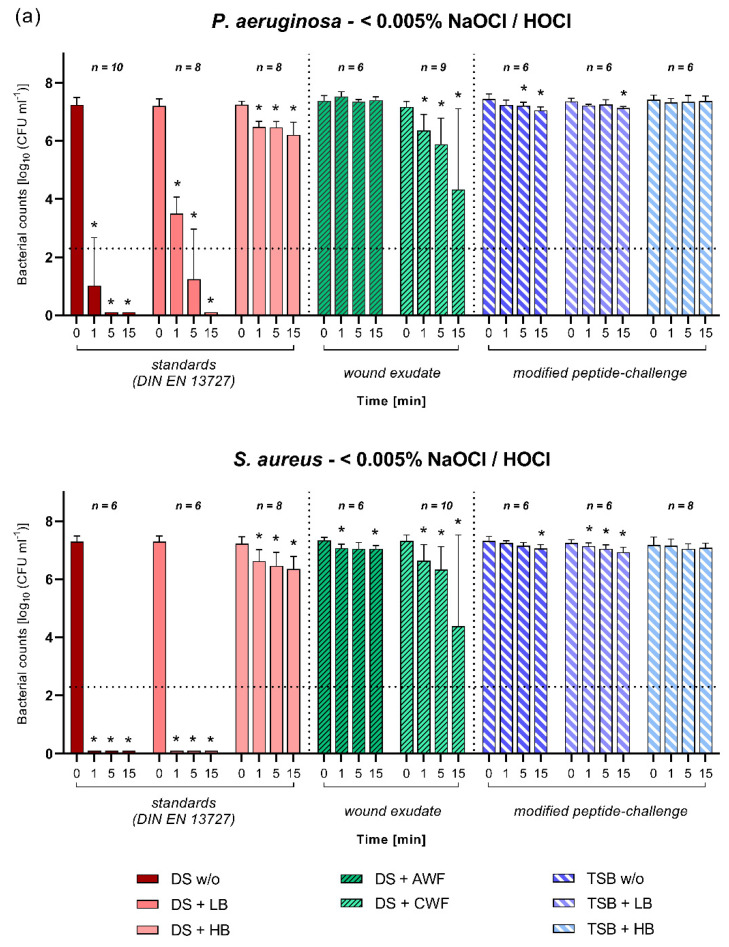

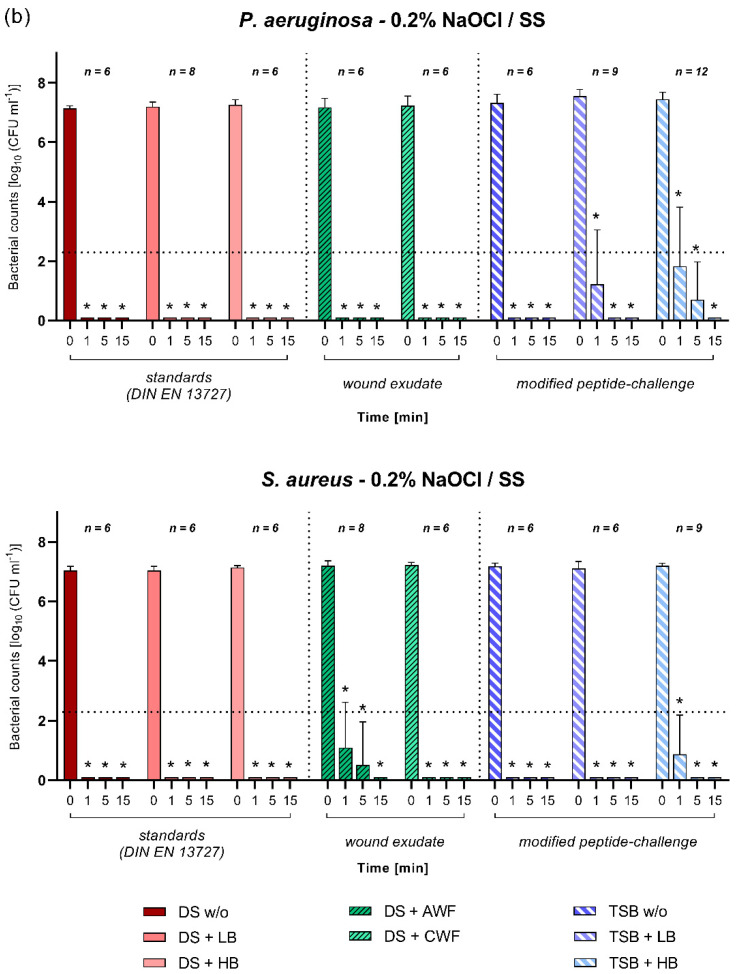

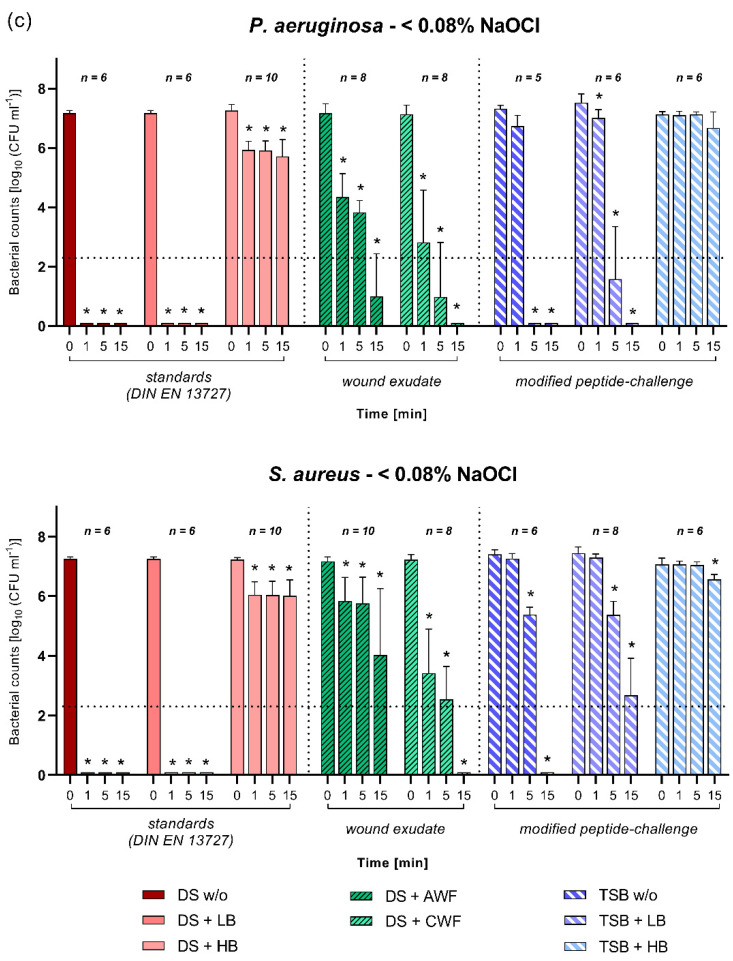
(**a**–**c**) Antimicrobial efficacy of hypochlorous wound irrigation solutions against *P. aeruginosa* and *S. aureus*. Three different hypochlorous formulations were tested: (**a**) <0.005% NaOCl/HOCl, (**b**) 0.2% NaOCl/SS, and (**c**) <0.08% NaOCl. Bacterial survival rates (in log_10_ CFU/mL) after 1, 5, and 15 min of exposure time under various challenge conditions and initial bacterial counts (0 min) are displayed. First section shows the standard DIN-EN-13727 conditions of no burden (DS w/o), low burden (DS + LB), and high burden (DS + HB) prepared in regular dilution solution (DS). Second section shows results when challenged with simulated wound conditions using human acute (DS + AWF) or chronic (DS + CWF) wound exudate in DS. Third section shows results for the modified peptide-challenge prepared in tryptone soy broth (TSB) with no burden (TSB w/o), low burden (TSB + LB), and high burden (TSB + HB). Values are expressed as mean ± SD (* *p* ≤ 0.05 vs. 0 min) and the dotted line represents a reduction in 5 log_10_ cfu/mL steps compared to initial bacterial counts.

NaOCl/HOCl (<0.005%) solution achieved a high efficacy and significantly reduced initial bacterial counts within 1 min (*p* ≤ 0.05) under the standards (DIN-EN-13727) that are unchallenged and low burden conditions against both *P. aeruginosa* and *S. aureus* (Figure 3a). For *S. aureus*, even a complete reduction in detectable bacterial counts was achieved. However, when a higher level of challenge and biological burden was introduced, no overall antimicrobial effect was detected. Under the standards of high burden condition, as well as the modified-peptide challenge (regardless of additional biological burden) and acute wound exudate, no effect was detected (Figure 3a). Under the challenged with chronic wound exudate <0.005%, NaOCl/HOCl demonstrated a significant reduction; however, only after 15 min of exposure and with a high standard deviation (P.a. −2.86 ± 2.78 and S.a. −2.93 ± 3.14 log_10_ steps; Figure 3a).

On the other hand, NaOCl/SS (0.2%) demonstrated an overall high antimicrobial efficacy achieving a general significant reduction in initial bacterial counts of >5 log_10_ steps within 1 min regardless of the challenge condition (Figure 3b). These results were consistent for both assessed bacterial species (*P. aeruginosa* and *S. aureus*). Against *P. aeruginosa*, complete reduction in detectable bacterial counts occurred for standard as well as human wound exudate conditions within 1 min. Only for the modified peptide-challenge a prolonged exposure of 5 min (low burden) and 15 min (high burden) were necessary to achieve complete reduction. For *S. aureus*, the acute wound exudate challenge and the modified peptide-challenge posed the necessity of prolonged exposure times for complete eradication (15 and 5 min, respectively; Figure 3b).

NaOCl (<0.08%) showed comparable results as <0.005% NaOCl/HOCl under the standard challenge conditions for both tested bacteria (Figure 3c). While a complete reduction in detectable counts was achieved within 1 min for unchallenged and low burden conditions, only a marginal reduction of about 1 log_10_ step could be observed under high burden challenge conditions. However, in contrast to NaOCl/HOCl (<0.005%) solutions, <0.08% NaOCl maintained a certain degree of efficacy when challenged with physiological simulated wound conditions. Under challenge with wound exudate, a significant high antimicrobial efficacy was achieved against *P. aeruginosa* within 15 min of exposure to acute wound exudate and within 5 min when challenged with chronic wound exudate (*p* ≤ 0.05). Under chronic exudate conditions, complete reduction in detectable counts was reached after 15 min of exposure. Against *S. aureus*, a similar pattern was observed under chronic wound exudate challenge, with complete reduction after 15 min of exposure. For acute wound exudate, an overall lower efficacy was observed, with the highest reduction rate achieved as 3.14 ± 2.23 log_10_ steps after 15 min (*p* ≤ 0.05; Figure 3c). For the modified peptide-challenge, a similar pattern as for the simulated wound conditions using wound exudate, with a certain degree of variation, could be observed (Figure 3c). However, in terms of the additional high burden in the modified peptide-challenge, no antimicrobial efficacy could be observed. Under high-peptide-challenge without additional burden, complete reduction was achieved within 5 min against *P. aeruginosa* and within 15 min against *S. aureus*. Under low burden peptide-challenge conditions, an increased exposure time of 15 min was necessary to completely reduce *P. aeruginosa*, while against *S. aureus*, the highest achieved reduction rate was 4.78 ± 1.24 log_10_ steps (*p* ≤ 0.05; Figure 3c).

### 3.3. Effect of Polyhexanide-Based Wound Irrigation Solution under Various Challenge Conditions

**Figure 4:**
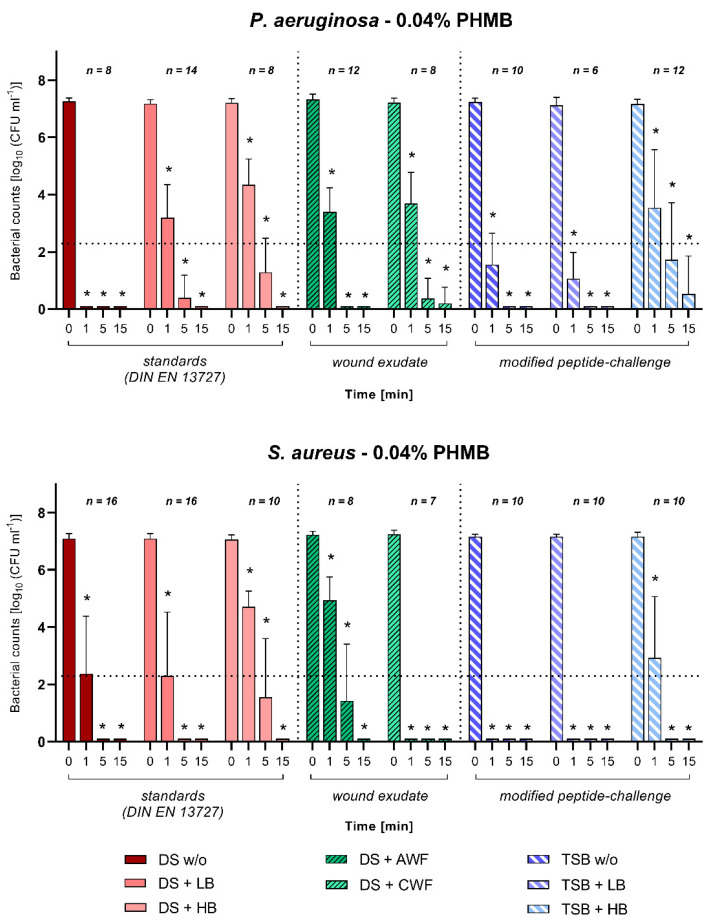
Antimicrobial efficacy of 0.04% polyhexanide-based (PHMB) wound irrigation solution against *P. aeruginosa* and *S. aureus*. Bacterial survival rates (in log_10_ CFU/mL) after 1, 5, and 15 min of exposure time under various challenge conditions and initial bacterial counts (0 min) are displayed. First section shows the standard DIN-EN-13727 conditions of no burden (DS w/o), low burden (DS + LB), and high burden (DS + HB) prepared in regular dilution solution (DS). Second section shows results when challenged with simulated wound conditions using human acute (DS + AWF) or chronic (DS + CWF) wound exudate in DS. Third section shows results for the modified peptide-challenge prepared in tryptone soy broth (TSB) with no burden (TSB w/o), low burden (TSB + LB), and high burden (TSB + HB). Values are expressed as mean ± SD (* *p* ≤ 0.05 vs. 0 min) and dotted line represents a reduction in 5 log_10_ cfu/mL steps compared to initial bacterial counts.

Overall, for polyhexanide (0.04% PHMB), heterogenous, highly effective results were observed against both bacterial species. Against both microorganisms, PHMB achieved a significant, high efficacy within 5 min the latest (*p* ≤ 0.05; Figure 4). Against *P. aeruginosa,* PHMB demonstrated a delayed reduction in bacterial counts under the standard conditions in line with the increase in biological burden. However, under low as well as high burdens, a complete reduction in detectable counts was achieved within 15 min. The same holds true for *S. aureus*. These results are comparable to the reductions observed under simulated wound conditions, whereas against both *P. aeruginosa* and *S. aureus*, complete reduction was obtained after 15 min. When challenged with the modified peptide-challenge and additional low or high burden, PHMB demonstrated a similar pattern of reduction against both pathogens. A significant reduction was achieved after 5 min of exposure and against *S. aureus* under no and low additional burden, even complete reduction was observed within 1 min (*p* ≤ 0.05; Figure 4). With an increasing biological burden (high burden), reduction in initial bacterial counts was reduced at the individual time-points and overall delayed over time.

### 3.4. Effect of Antiseptics (OCT/PE and PVP-IOD) under Various Challenge Conditions

**Figure 5:**
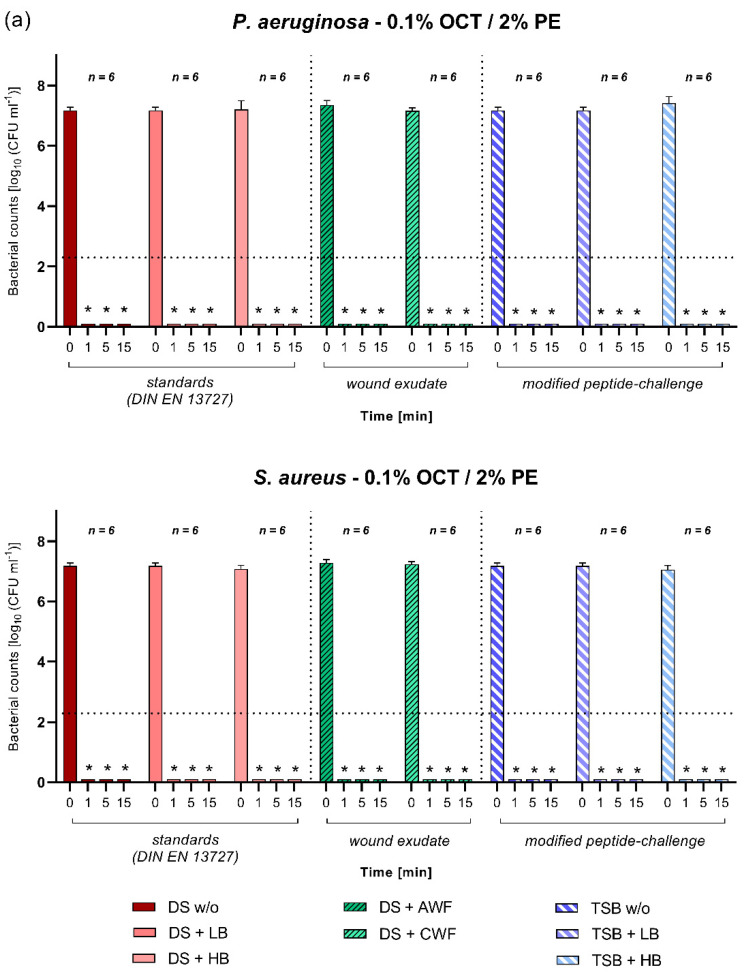

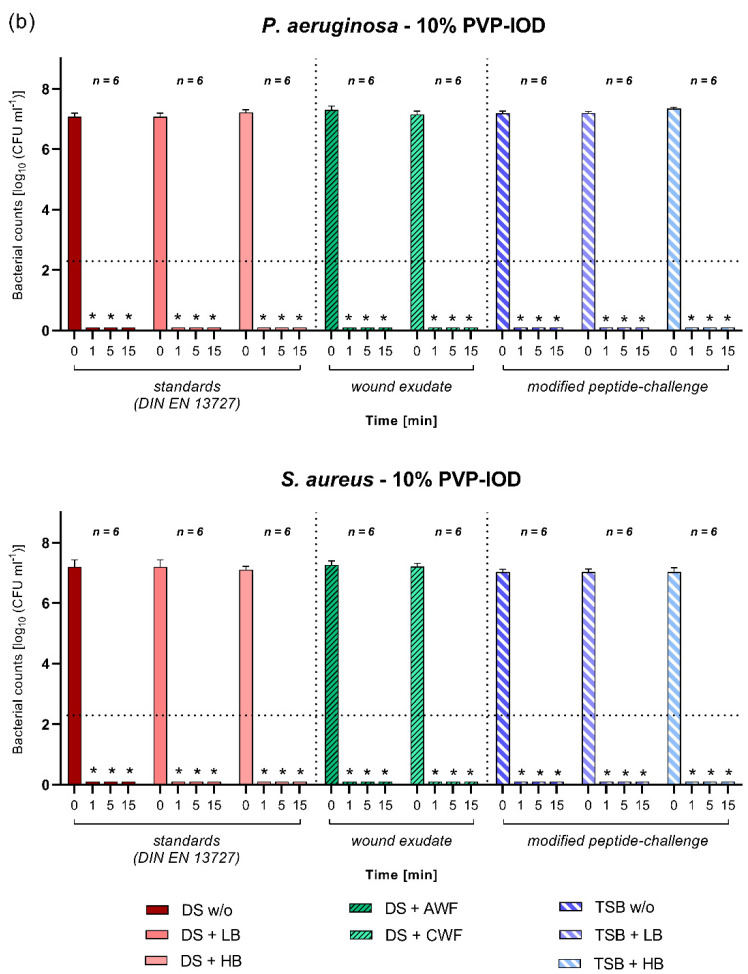
(**a**,**b**) Antimicrobial efficacy of (**a**) 0.1% octenidine/2% phenoxyethanol (OCT/PE) and (**b**) 10% povidone-iodine (PVP-IOD) antiseptics against *P. aeruginosa* and *S. aureus*. Bacterial survival rates (in log_10_ CFU/mL) after 1, 5, and 15 min of exposure time under various challenge conditions and initial bacterial counts (0 min) are displayed. First section shows the standard DIN-EN-13727 conditions of no burden (DS w/o), low burden (DS + LB), and high burden (DS + HB) prepared in regular dilution solution (DS). Second section shows results when challenged with simulated wound conditions using human acute (DS + AWF) or chronic (DS + CWF) wound exudate in DS. Third section shows results for the modified-peptide challenge prepared in tryptone soy broth (TSB) with no burden (TSB w/o), low burden (TSB + LB), and high burden (TSB + HB). Values are expressed as mean ± SD (* *p* ≤ 0.05 vs. 0 min) and dotted line represents a reduction in 5 log_10_ cfu/mL steps compared to initial bacterial counts.

The antiseptics tested here, octenidine/phenoxyethanol (OCT/PE, 0.1%/2%) and povidone-iodine (PVP-IOD, 10%), demonstrated a significant (*p* ≤ 0.05) and high efficacy under all investigated challenge conditions (Figure 5a,b). This result occurred regardless of the investigated microorganism (*S. aureus* or *P. aeruginosa*) and the exposure time. Under all challenge conditions, OCT/PE and PVP-IOD managed a complete reduction in detectable microorganism within 1 min of exposure.

### 3.5. Agreement and Deviation between Challenge Conditions Reflecting the Wound Environment (AWF and CWF)

Using Bland-Altmann (B&A) analysis and plots, the magnitude and significance of differences between test challenge conditions and the simulated wound environment (represented by AWF and CWF) could be investigated. Thereby, results obtained under the challenge with human AWF or CWF were considered as comparison standard, assuming that they represent the conditions in which antimicrobials best interact with in an actual wound micro-environment. An optimal standardized test condition should not significantly deviate from the comparison standard and show no significant bias from the line of equality (y = 0). Results of the efficacy testing for <0.08% NaOCl were best suited for agreement/difference analyses using B&A analysis, which is the reason why they were used in an exemplary way here (other efficacy results showed complete reduction, no relevant reduction at all or clear deviations in methodological results). Evidence of the general difference between the standards challenge (DS + LB and DS + HB) and the modified peptide-challenge (TSB + LB and TSB + HB) is demonstrated via B&A analyses and plots in Supplementary Figure S1.

**Figure 6:**
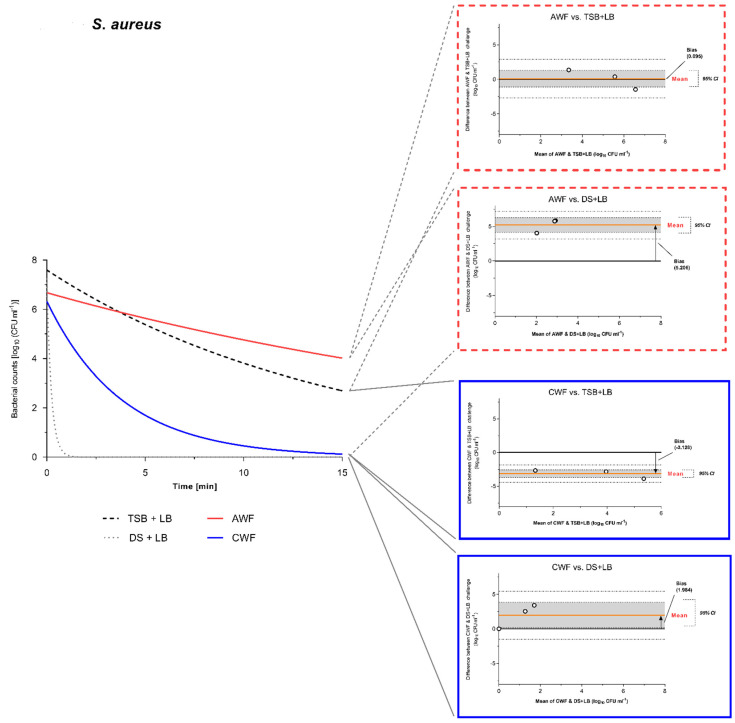
Bland-Altman (B&A) plot comparing bacterial reduction results against *S. aureus* under AWF challenge to TSB + LB and DS + LB. Best fitted curves of the reduction in bacterial counts over the course of 15 min exposure time for AWF (red line), CWF (blue line), DS + LB (dotted line), and TSB + LB (dashed line) are depicted on the left. Bland-Altman plots for the comparison between different challenge methods are shown on the right (dashed red frame for comparisons against AWF; blue solid line against CWF). Red line labeled “mean” marks the mean bias between the compared methods and deviation from the line of equality (y = 0). Grey area around the mean marks the 95% confidence interval. If the line of equality (y = 0) is enclosed in the 95% CI, no significant difference between methods can be postulated, if the line is excluded from the 95% CI, the methods provide significantly different results. The bias is stated numerically in the form of units measured (here log_10_ CFU/mL).

**Figure 7:**
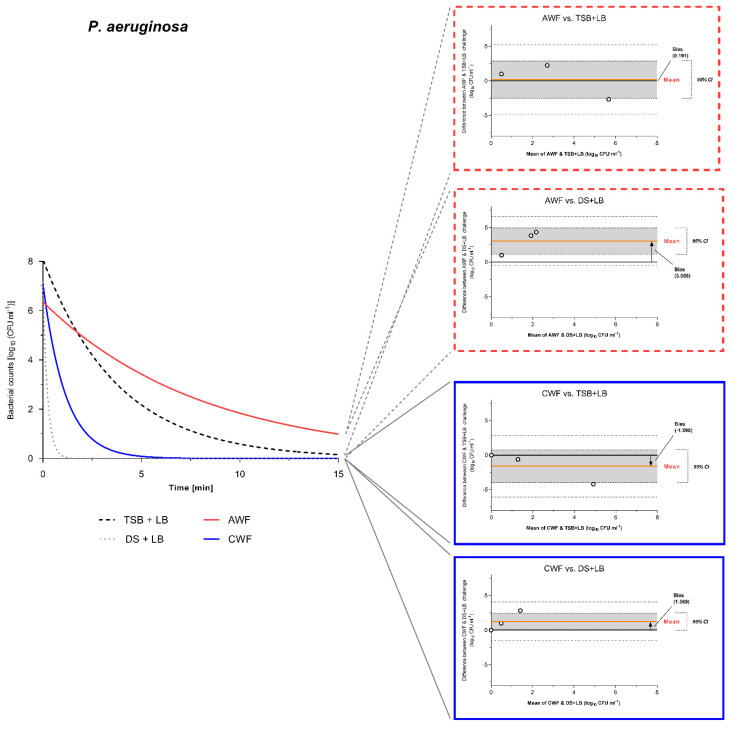
Bland-Altman (B&A) plot comparing bacterial reduction results against *P. aeruginosa* under AWF challenge to TSB + LB and DS + LB. Best fitted curves of the reduction in bacterial counts over the course of 15 min exposure time for AWF (red line), CWF (blue line), DS + LB (dotted line), and TSB + LB (dashed line) are depicted on the left. Bland-Altman plots for the comparison between different challenge methods are shown on the right (dashed red frame for comparisons against AWF; blue solid line against CWF). Red line labeled “mean” marks the mean bias between the compared methods and deviation from the line of equality (y = 0). Grey area around the mean marks the 95% confidence interval. If the line of equality (y = 0) is enclosed in the 95% CI, no significant difference between methods can be postulated, if the line is excluded from the 95% CI, the methods provide significantly different results. The bias is stated numerically in the form of units measured (here log_10_ CFU/mL).

Comparing the modified peptide-challenge conditions with low burden (TSB + LB; 0.3% BSA) to the simulated acute wound conditions (AWF), no significant bias between the methods can be observed with a mean bias of 0.095 log_10_ steps (95% CI [−1.10, 1.29]) for *S. aureus* (Figure 6) and a mean bias of 0.191 log_10_ steps (95% CI [−2.50, 2.88]) for *P. aeruginosa* (Figure 7). This shows a high degree of agreement between the methods. In terms of the current standard challenge conditions with low burden (DS + LB; 0.3% BSA) compared to AWF, the methods show a significant bias between the method results with a mean bias of 5.206 log_10_ steps (95% CI [4.13, 6.28]) for *S. aureus* (Figure 6) and a mean bias of 3.058 log_10_ steps (95% CI [1.17, 4.94]) for *P. aeruginosa* (Figure 7). Therefore, the standard challenge condition overestimates the efficacy of the tested antiseptics by 5.206 and 3.085 log_10_ steps, respectively. Moreover, this becomes clear by observing the bar graph depictions in Figure 3c. In terms of high burden challenges (HB; 3% BSA and 3% SE), both challenge conditions (DS + HB and TSB + HB) demonstrated a significant methodological difference in efficacy results compared to AWF against both tested microorganisms (Supplementary Figures S2a and S3a). However, the modified peptide-challenge with no additional burden (TSB w/o; no BSA, no SE), showed no significant methodological difference regarding efficacy results compared to AWF (Supplementary Figures S2b and S3b). Generally, the modified peptide-challenge (especially TSB + LB and TSB w/o) demonstrated a higher degree of methodological agreement with the comparison standard of an acute wound micro-environment than the current standard challenge (DS w/o, DS + LB, and DS + HB).

For the methodological comparison of test challenge conditions against the simulated chronic wound conditions (CWF), results of the B&A analyses proved heterogenous. The modified peptide-challenge in tests with *S. aureus* significantly underestimated the efficacy of the antimicrobial (TSB w/o-mean bias of −2.231 log_10_ steps, 95% CI [−4.33, −0.14] (Supplementary Figure S2b) and TSB + LB-mean bias of −3.128 log_10_ steps, 95% CI [−3.68, −2.58] (Figure 6)) compared to CWF. However, the standard challenge (DS + LB) significantly overestimated the efficacy with a mean bias of 1.984 log_10_ steps, 95% CI [0.12, 3.84] (Figure 6). On the contrary, against *P. aeruginosa,* the modified peptide-challenge (TSB w/o and + LB) demonstrated no significant bias (TSB w/o-mean bias of −0.978 log_10_ steps, 95% CI [−4.20, 2.24] (Supplementary Figure S3b) and TSB + LB-mean bias of −1.598 log_10_ steps, 95% CI [−3.98, 0.78] (Figure 7)). Here, the standard challenge again significantly overestimated the antimicrobials efficacy (DS + LB-mean bias of 1.269 log_10_ steps, 95% CI [0.07, 2.47] (Figure 7). Ultimately, of course, for the high burden challenges (HB; 3% BSA and 3% SE), both challenge conditions (DS + HB and TSB + HB) demonstrated a significant methodological difference in efficacy results compared to CWF against both tested microorganisms, underestimating the antimicrobial efficacy (Supplementary Figures S2a and S3a).

## 4. Discussion

In everyday clinical practice, clinicians rely on the efficacy profiles of antimicrobial substances, solutions, and dressings obtained as part of the product approval process for the indicated use as skin and wound antiseptics. However, no universal standard exists for the evaluation of the antimicrobial product group “wound irrigation solutions and topical antiseptics”. Therefore, manufacturers and researchers in Europe mostly refer to DIN-EN-13727 [8] for antimicrobial efficacy evaluation and product approval. This specific standard is designed and designated for chemical disinfectants and antiseptics to be used on surfaces or intact skin (products for surgical and/or hand disinfection and/or washing). Therefore, the test conditions described in the standards are not designed to reflect the clinical use of substances in acute or chronic wounds, nor in wound cavities or on mucous membranes.

The human wound micro-environment differs greatly between individuals and phases of the healing process, with various influential factors, such as cellular and extracellular composition, pH value, underlying pathophysiology or bacterial load and diversity [12,13]. An increased protein concentration has proven to be one of the most influential (efficacy-inhibiting) parameters in previous studies [14,15]. To account for this factor, within the scope of DIN-EN-13727, different challenge substances (bovine albumin and/or sheep erythrocytes) can be added to the test setup, aiming to approximate the “field of application” of the product and simulate clinical conditions (e.g., a complex wound micro-environment). However, several problems arise with the current standard practice. The use of an “organic load” is, whilst recommended, not mandatory in the standards and free choice is provided, whether a wound irrigation solution or antiseptic is tested with or without potentially interfering challenge substances. If a challenge substance is used, two different concentrations of added protein (bovine albumin), differing by a factor of 10 (Figure 2), can be selected in the DIN-EN-13727, representing a lower or higher challenge burden. In the case of “higher burden”, 3% sheep erythrocytes are additionally added to represent blood-contaminated “dirty” conditions.

Whilst the general consideration of varying challenge conditions to approximate the efficacy in a clinical setting is valid, the execution within the current standards is insufficient. Although a standardized test should ideally be easy to perform, error-tolerant, and broadly applicable, it also needs to adequately represent the circumstances and conditions it is supposed to assess. In this case, the influence exerted by the complex wound micro-environment on the antimicrobial efficacy of tested substances as well as the specific configuration and mode of action of the tested substance itself. The aim should be to investigate a substance in as close a simulation of the clinical conditions in which it is used in reality as possible. This raises another difficulty, as the exact composition and interactions of the interindividually heterogenous wound micro-environment are still scarcely decoded in detail. Therefore, investigations on antimicrobial product interactions with the actual human wound micro-environment have only emerged within the past few years, but clearly demonstrated the urgent necessity to more carefully account for this influence on antimicrobial performance [3,4,5].

In the present in vitro analyses, the wound micro-environment was therefore simulated using human acute and chronic wound exudate (AWF and CWF) as reference standard for challenge conditions. These were compared to the current standard test settings (DIN-EN-13727) and a modified peptide-challenge with a higher baseline concentration of peptides compared to the standard challenge substances. The aim was to evaluate the level of agreement or difference between established standard test scenarios and the wound micro-environment regarding efficacy outcomes and to observe the performance of a refined approach for a potential challenge substance (modified peptide-challenge).

The used physiological wound challenges AWF and CWF were pooled patient samples, which may vary intra- and inter-individually in their organic and biochemical composition due to their wound-specific diversity (Table 2). Due to this variability and the limited availability of human wound exudate, the actual exudate cannot generally function as a challenge substance for extensive standardized testing. Therefore, standardizable artificial challenge substances, which adequately simulate or at least as close as possible approximate the clinical conditions and interactions between the wound micro-environment and substance are sought. In particular, exudates of infected wounds contain blood components [14] as well as necrotic tissue fragments of extracellular matrix (ECM) degradation, cell debris, fibrin remnants, and numerous peptides generated by proteolytic processes [16]. This degraded peptide-rich baseline environment forms the rational for the modified peptide-challenge used here (TSB), to simulate the peptide content in wound exudates not covered by the macromolecular proteins (albumin) added in the current standards, whereas the challenge conditions in the test setup are reproducible due to the standardized peptide/protein concentrations.

When comparing the three challenge conditions (standard, wound exudate, modified peptide-challenge), regarding the total protein concentration, the three levels of standard challenge showed the expected protein concentration difference to the factor 10 (Figure 2) and the modified peptide-challenge matched the total protein concentration with slightly higher concentrations on all three levels attributable to the higher peptide baseline. The high burden (HB; 46.17 mg/mL) showed a slightly lower total concentration than the human undiluted AWF pool (AWF; 51.78 mg/mL), which can be attributed to the proportion of peptides contained in AWF. In general, the overall protein concentrations of AWF are in line with previously described concentrations, albeit slightly higher [17,18,19]. For CWF, a lower total protein concentration was measured in this study, which can be attributed to the necessary sampling and processing methodology resulting in a dilution of the initial samples to approximately 37% of the concentration to be expected in a chronic wound bed. When the observed mean concentration (CWF; 15.87 mg/mL) is extrapolated to 100% (~42.89 mg/dL), the CWF concentration resembles total protein concentrations measured in earlier studies [4,19] as well as in the comparative challenge substances in this study. However, due to this dilution, results for CWF need to be interpreted with extra care.

Based on the sole concentration of total protein content, the high burden level of both challenges should reflect the clinical wound micro-environment best. Alternatively, the obtained results and analyses of agreement between challenges demonstrated that for highly concentrated antiseptics (OCT/PE, PVP-IOD; Figure 5) and antimicrobial irrigation solutions with highly concentrated additives (PHMB, NaOCl/SS; Figure 3b and Figure 4), the overall concentration (as well as composition) of the challenge substances showed little relevance. More specifically, for antiseptics OCT/PE and PVP-IOD, no protein error was observed, neither under physiological conditions (AWF and CWF), nor simulated challenges, testifying a high and undisturbed efficacy in the wound micro-environment. Comparable results can be observed for antimicrobial wound irrigation solutions with high active agent concentrations (PHMB and NaOCl/SS), whereas efficacy was fairly similar between the two challenge conditions. Particularly for PHMB, a concentration-dependent decrease in efficacy can be observed for higher burden; however, both the standard and the modified peptide-challenge represent the AWF and CWF reference equally well (Figure 4). Therefore, total protein concentration plays a certain relevant role in efficacy inhibition for some substances. However, these results not only demonstrate that substance-dependent challenge scenarios need to be considered, as “one-fits-all” approaches are discouraged by the obtained results. Moreover, it is underpinned that the total protein concentration in a challenge substance alone is not decisive for a potential loss of efficacy, but also, its composition.

In particular, this becomes clear in the class of hypochlorous wound irrigation solutions, which have recently been increasingly introduced into the market under various product names with different constitutions (Table 1). Figure 3a demonstrates the tremendous overestimation of the antimicrobial efficacy for wound irrigation solutions containing <0.005% NaOCl/HOCl in the case of the current standard evaluation under no (DS w/o) and low burden (DS + LB) compared to the simulated physiological wound environment represented by AWF and CWF. While complete reduction within 1 min is reached under standard conditions (for DS w/o and DS + LB), microorganisms show no relevant reduction after 15 min of exposure to AWF and CWF. Therefore, the more comparable results under the modified-peptide challenge (TSB w/o and TSB + LB) demonstrate that the composition of the challenge substance matters more than the mere total concentration, since DS w/o (0.038 mg/mL) and DS + LB (0.398 mg/mL) only differ slightly in total protein concentrations compared to TSB w/o (0.176 mg/mL) and TSB + LB (0.536 mg/mL). This fact is demonstrated even more distinctively by the results obtained for <0.08% NaOCl (Figure 3c). Here, the higher concentrated antimicrobial irrigation solution exerts an overall better efficacy; however, it is still highly overestimated by the no and low burden challenge of the DIN-EN-13727 standard compared to the physiological reference AWF and CWF. Moreover, the modified peptide-challenge demonstrates a better agreement with the simulated clinical reality. This is statistically reinforced by the performed Blunt-Altman analysis that shows a significant bias and therefore difference in results obtained for the standard challenge conditions, regardless of the degree of burden (DS + LB—Figure 6 and Figure 7; DS + HB—Supplementary Figures S2a and S3a), and the AWF/CWF reference. Therefore, the standard no and low burden significantly overestimate the antimicrobials performance, while the high burden significantly underestimates their efficacy. On the contrary, the modified peptide-challenges no and low burden (TSB w/o—Supplementary Figures S2b and S3b; TSB + LB—Figure 6 and Figure 7) show no significant bias to the reference method with AWF and CWF and therefore a high methodological agreement. The only exception is the comparison of CWF vs. TSB + LB for *S. aureus* (Figure 6), whereas TSB + LB significantly deviate from the results for CWF, which can most likely be attributed to the diluted CWF samples. The high burden challenge conditions (DS + HB and TSB + HB) described in DIN-EN-13727 do not reflect the physiological wound micro-environment (AWF and CWF): The albumin/sheep erythrocyte combination underestimated the wound irrigation solutions efficacy compared to AWF and CWF (Figure 3a,c).

Most likely, the addition of peptides to the challenge substance is the reason for the improved simulation of the clinical conditions by the low burden of the modified peptide-challenge (TSB + LB), which in terms more adequately approximates the acute and chronic wound micro-environment with its higher degree of proteolytic degradation. Peptides, such as peptones, have shown a significant influence on the efficacy of wound irrigation products in earlier studies [20,21,22]. Therefore, the highly reactive chlorine-based species are more likely to interact with the exposed amino acid aspects of peptides than the more complex structure of macro proteins, resulting in a faster consumption of active agents by side-interactions rather than wanted interaction with microorganisms [23,24]. These specific considerations underline the crucial relevance to approximate artificial challenge substances to the actual wound micro-environment. On the other hand, these interactions would not be considered in efficacy evaluations resulting in biased and non-translatable antimicrobial efficacy profiles.

Overall, these results propose that the modified peptide-based challenge conditions (TSB w/o and TSB + LB) better represent and reflect the physiological interaction and efficacy of wound irrigation solutions than the current standard challenge conditions. Therefore, this modified peptide-challenge could be defined as a prescribed baseline challenge condition in a future revised specific set of standards, adapted to the group “antimicrobial wound irrigation solutions and antiseptics”. Naturally, the singular addition of peptides in standardized challenge procedures still does not reflect the interactions and challenges within the wound-microenvironment in its complexity. Nevertheless, it represents a simplified approach, which presents superior standards compared to the current established standards in reflecting and approximating the reality, as demonstrated by the degree of agreement with the comparison standard reflected by AWF and CWF. Whether the addition of further potential challenge additives or conditions (such as cytokines or specified pH ranges) would introduce additional benefits regarding a translational simulation of the wound micro-environment is to be determined in future studies and validated by different laboratories and centers as well as in more complex approaches (such as 3D models, human biofilm setups, and/or animal studies). Nevertheless, the results presented here demonstrate a relevant step toward refining the pre-clinical evaluation of antimicrobials in wound care.

## 5. Conclusions

In summary, the closer a test method for efficacy profile evaluation is adapted to the subsequent field of application, the more reliable a statement can be made regarding its efficacy in (clinical) practice. With respect to antimicrobial wound solutions, the currently established standards (DIN-EN-13727) do not adequately meet these criteria for all categories of antimicrobial irrigation solutions and antiseptics. As demonstrated here, current challenge conditions in the standards, designed to simulate the (clinical) reality, drastically under- or over-estimate the efficacy of a whole category of wound antimicrobials (hypochlorous-based solutions) compared to a human wound exudate reference standard. Therefore, it is necessary to develop and establish adapted standardized test conditions in accredited laboratories or to create new standards for wound-specific antimicrobial irrigation solutions and antiseptics. Therefore, relevant “close to reality” influences and interactions of the wound micro-environment as well as specific considerations regarding active agents in substances should be considered. As a first step, the broad introduction of the performed protein/peptide modification in the modified peptide-challenge into standardized testing should be considered, as it demonstrated a significantly better agreement with the results obtained under simulated physiological wound conditions (AWF and CWF). This would allow for testing the antimicrobial potential of a substance or solution in a more representable artificial wound micro-environment.

## Figures and Tables

**Table 1:** Overview of investigated antimicrobials and antiseptics. Substances are categorized in antiseptics and antimicrobials based on common consent and manufacturer specifications. Test solution, product name, manufacturer, active agent composition, and known accreditation are stated (MP: Medical product; MD: Medical device).

Antimicrobials and Antiseptics					
Test Solution		Product	Manufacturer	Active Agent Composition	Accredation
Antiseptic	OCT/PE	Octenisept^®^	Schülke & Mayr GmbH (Norderstedt, Germany)	1 g/L (0.1%) octenidin dihydrochloride, 20 g/L (2%) 2-phenoxyethanol	MP
	PVP-IOD	Betaisodona^®^	Mundipharma GmbH (Frankfurt am Main, Germany)	100 g/L (10%) povidone-iodine	MP
Antimicrobial	PHMB	Lavasorb^®^	Fresenius Kabi AG (Bad Homburg, Germany)	0.4 g/L (0.04%) polyhexanide, 0.02 g/L (0.002%) macrogolum 4000	MD (II b)
	NaOCl/HOCl	Granudacyn^®^	Mölnlycke Health Care GmbH (Düsseldorf, Germany)	each <0.005% NaOCl and HOCI	MD (II b)
	NaOCl	Lavanox^®^	Serag Wiessner GmbH & Co KG (Naila, Germany)	<0.08% NaOCl	MD (II a)
	NaOCl/SS	ActiMaris^®^forte	ActiMaris AG (Appenzell, Switzerland)	3% sea salt, 0.2% NaOCl	MD (II b)

**Table 2:** Patient demographics of included wound exudate pools. Demographic data including age, gender, and wound etiology are summarized for acute wound exudate (*n* = 6) and chronic wound exudate pools (*n* = 31).

Patient Demographics		
	Acute (AWF)	Chronic (CWF)
*Characteristic*	Median (Range)	Median (Range)
	n (%)	n (%)
	***n* = 6**	***n* = 31**
**Age (years)**	64 (49–77)	68 (42–88)
**Gender**		
Male	4/6 (66.7)	16/31 (51.6)
Female	2/6 (33.3)	15/31 (48.4)
**Wound etiology**		
Lower leg amputation	2/6 (33.3)	
Femoral thrombendarteriectomy	3/6 (50.0)	
Augmentation mammoplasty	1/6 (16.7)	
PAD ^†^		6/31 (19.4)
CVI ^‡^		8/31 (25.8)
Ulcus cruris mixtum		4/31 (12.9)
Lipedema		1/31 (3.2)
LEP *		2/31 (6.5)
Vasculitis		4/31 (12.9)
Necrobiosis lipoidica		2/31 (6.5)
Wound healing disorder (post-surgery)		2/31 (6.5)
Pyoderma gangraenosum		2/31 (6.5)

^†^ Peripheral arterial disease. ^‡^ Chronic venous insufficiency. * Lupus erythematosus panniculitis.

**Table 3:** Experimental challenge conditions in the quantitative suspension method (QSM). Composition of the experimental setups in the quantitative suspension method using different challenge substances and bacterial suspensions to which the test solution (antimicrobial/antiseptic agent) is added.

Setup Challenge Conditions								
Volume Fraction	DIN-EN-13727 Challenges			Wound Challenges		Peptide/Protein Challenges		
	*DS w*/*o*	*DS + LB*	*DS + HB*	*AWF*	*CWF*	*TSB w*/*o*	*TSB + LB*	*TSB + HB*
**100 µL**	BS-DS	BS-DS	BS-DS	BS-DS	BS-DS	BS-TSB	BS-TSB	BS-TSB
**100 µL**	H_2_O d.d.	0.3% BSA	3% BSA, 3% SE	AWF	CWF	H_2_O d.d.	0.3% BSA	3% BSA, 3% SE
**800 µL**	TS	TS	TS	TS	TS	TS	TS	TS

(TS: Test solution; DS: Diluent solution; BS-TSB: Bacterial suspension in tryptone soy broth (TSB); BS-DS: Bacterial suspension in diluent solution; LB: Low burden; HB: High burden; AWF: Acute wound exudate; CWF: Chronic wound exudate; H_2_O: Water; BSA: Bovine serum albumin; SE: Sheep erythrocytes).

## Data Availability

The data presented in this study are available on request from the corresponding author.

## Supplementary Materials

The following supporting information can be downloaded at: https://www.mdpi.com/article/10.3390/biomedicines10112751/s1, Figure S1: Depiction of the method comparison (Bland-Altman analysis and plot) between DS + LB vs. TSB + LB as well as DS + HB vs. TSB + HB for *S. aureus* and *P. aeruginosa*. Figure S2: Bland-Altman analysis and plot for methodological comparison of AWF and CWF vs. DIN-EN-13727 high burden (DS + HB; (a)) and modified peptide-challenge without additional burden (TSB w/o; (b)) for efficacy tests with *S. aureus*. Figure S3: Bland-Altman analysis and plot for methodological comparison of AWF and CWF vs. DIN-EN-13727 high burden (DS + HB; (a)) and modified peptide-challenge without additional burden (TSB w/o; (b)) for efficacy tests with *P. aeruginosa*.
