# Supplementary material for: Composition of Challenge Substance in Standardized Antimicrobial Efficacy Testing of Wound Antimicrobials Is Essential to Correctly Simulate Efficacy in the Human Wound Micro-Environment
Source: Biomedicines. 2022 Oct 29;10(11):2751. doi: 10.3390/biomedicines10112751 (PMC9687328; doi:10.3390/biomedicines10112751)
Supplement: Supplementary file 1 [file biomedicines-10-02751-s001.zip › biomedicines-1974717-supplementary.pdf]

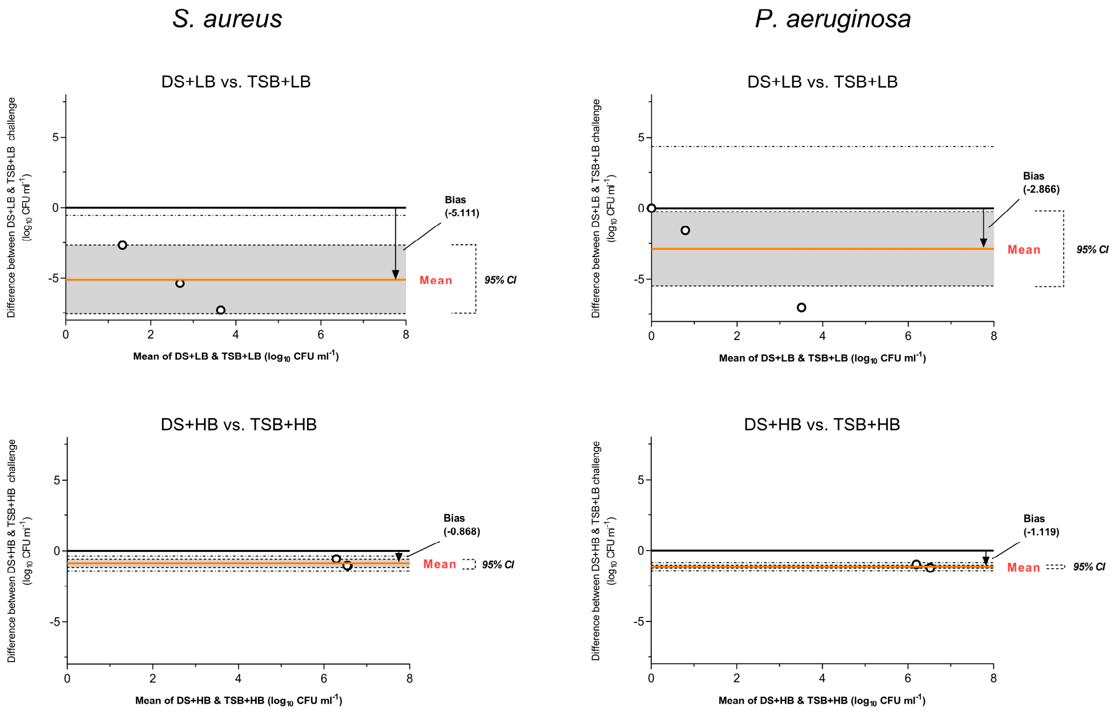

Figure S1. Depiction of the method comparison (Bland-Altman analysis and plot) between DS + LB vs. TSB + LB as well as DS + HB vs. TSB + HB for *S. aureus* and *P. aeruginosa*.

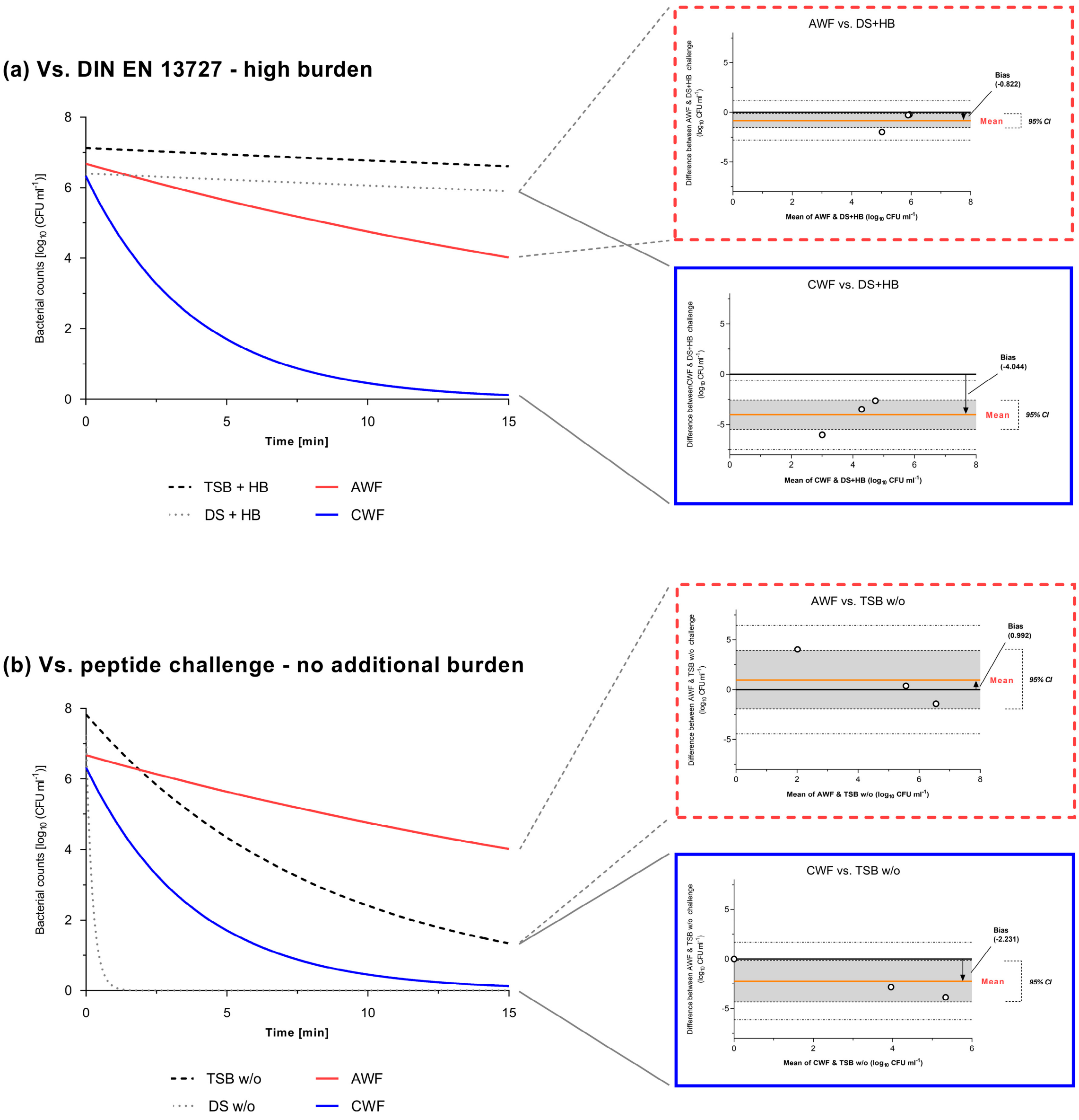

Figure S2. Bland-Altman analysis and plot for methodological comparison of AWF and CWF vs. DIN-EN-13727 high burden (DS + HB; (a)) and modified peptide-challenge without additional burden (TSB w/o; (b)) for efficacy tests with *S. aureus*.

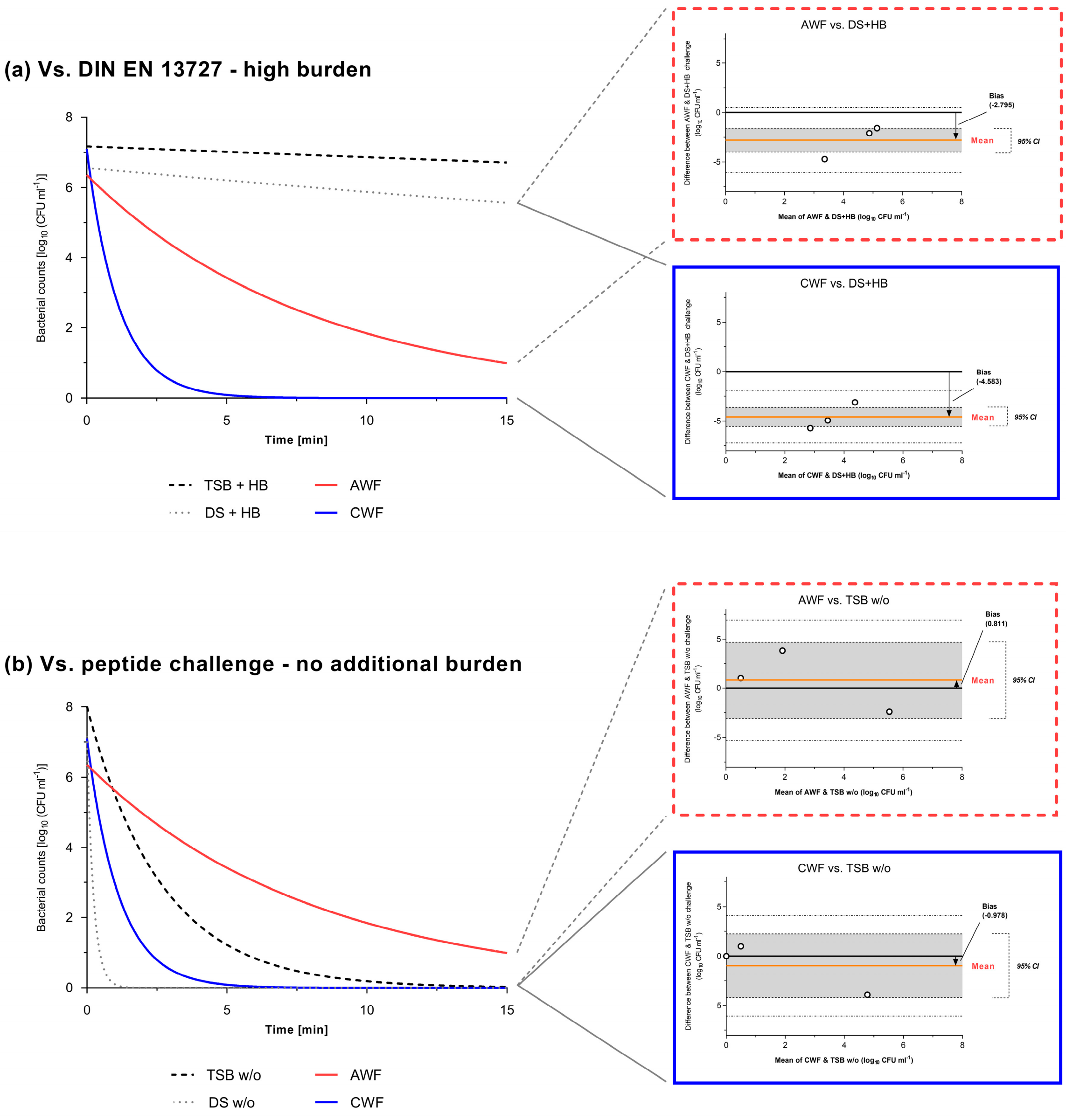

Figure S3. Bland-Altman analysis and plot for methodological comparison of AWF and CWF vs. DIN-EN-13727 high burden (DS + HB; (a)) and modified peptide-challenge without additional burden (TSB w/o; (b)) for efficacy tests with *P. aeruginosa*.
